# Reduction of inappropriate prescriptions in older adults through the support of Asynchronous Geriatric Counseling Online (AGAlink): Implemented in primary care

**DOI:** 10.1371/journal.pone.0258414

**Published:** 2021-11-17

**Authors:** Annia Marisol Avalos-Mejia, Juan Carlos García-Cruz, Jorge Escobedo de la Peña, Osvaldo Garrido-Acosta, Teresa Juárez-Cedillo

**Affiliations:** 1 Unidad de Investigación Epidemiológica y en Servicios de Salud, Área Envejecimiento, Centro Médico Nacional Siglo XXI. Instituto Mexicano del Seguro Social (Actualmente comisionada en la Unidad de Investigación en Epidemiológica Clínica, Hospital General Regional Núm. 1 Dr. Carlos Mac Gregor Sánchez Navarro, IMSS), Ciudad de México, México; 2 División de Medicina Geriátrica, Departamento de Medicina., Hospital de Especialidades, Centro Médico Nacional Siglo XXI, IMSS, Ciudad de México, México; 3 Unidad de Investigación en Epidemiológica Clínica, Hospital General Regional Núm. 1 Dr. Carlos Mac Gregor Sánchez Navarro, IMSS, Ciudad de México, México; 4 Facultad de Estudios Superiores Zaragoza, UNAM, Estado de México, México; University of South Australia, AUSTRALIA

## Abstract

**Background/Aim:**

Medication prescription is a fundamental component in the care of the elderly. Several characteristics of aging and geriatric medicine affect prescriptions for these people and make the selection of drug therapy a difficult and complex process. The objective of this study is to develop a geriatric portal for asynchronous online counseling (AGAlink) for use by physicians specializing in family medicine to reduce medication problems among older adult patients in the first level of care.

**Method:**

A qualitative study was carried out in the first level of care at the Mexican Institute of Social Security (IMSS), 31 family doctors were interviewed to identify attitudes, preferences about the use of the AGAlink geriatric portal, as well as their recommendations for the implementation of this tool in their daily practice. For the analysis of the data obtained, a qualitative thematic content analysis was used.

**Results:**

90% of the physicians used the geriatric portal outside office hours without the need for the patient to be present. The perception of the physician towards the use of the AGAlink geriatric portal was favorable, provided relevant information and had several positive effects on the process of care for medical prescription. The barriers identified to accept the change in medication were not having the proposed therapeutic option, lack of any laboratory analysis, continuing to consider their experience for the prescription of the medication.

**Conclusions:**

The AGAlink geriatric portal was a tool that was well received by physicians who expressed a positive attitude, considered an investment of a short time that allowed them to update and learn about strategies to reduce the prescription problems presented among the elderly population. However, the main barrier was the use of technology, especially in the doctors with more seniority in the service.

## Introduction

Medication prescription is a fundamental component in the care of the elderly. Several characteristics of aging and geriatric medicine affect prescriptions for these people and make the selection of drug therapy a difficult and complex process [1]. Interindividual variability in health, disease, and disability conditions increases considerably with age. Although there is a growing number of elderly, healthy people, there is also a high number of those who are vulnerable and fragile presenting a limited physiological reserve, a reduction in homeostasis, dysregulations in the immune and inflammation mechanisms, various chronic diseases, which makes them susceptible to polypharmacy [2]. Evidence suggests that the use of drugs in older people is often inappropriate, and can cause morbidity, representing a clinical and economic burden for both patients and society. Therefore, inappropriate prescribing among the elderly has become a major public health problem around the world [3].

A number of methods have been developed to evaluate appropriate prescription drugs for older people. The Beers criteria are commonly used and have been widely validated. These criteria are central to a list of 21 drugs that are potentially unsuitable for use by older people [4,5]. Two instruments were validated in Spain for the detection of Inappropriate Prescription in the elderly, for its acronym in English STOPP / START, the first is the tool for screening potentially inappropriate prescriptions and the second is the tool to detect errors by omission. Potentially inappropriate medications for the elderly are associated with lower quality-of-life-related health status and higher utilization of health services [6–8].

In response to the high and sustained frequency of inappropriate prescribing, it is recommended to carry out interventions based on methods that make it possible to reduce the possible inappropriate prescription of drug, such as education of medical personnel for the application of the Beers criteria, software development for inappropriate prescription query, computational alerts, etc., however these strategies have not yielded good results due to lack of response, sample size, etc. [9–11].

Recently, it has been reported that computer-aided therapeutic decision support has significantly improved medical performance and clinical practice in approximately two-thirds of cases. However, so far in our country this type of tool has not been tested, much less in the field of primary care, and it is not clear whether the implementation of this type of support would be effective in improving prescribing, in the environment that characterizes family medicine units, where the pace of work is fast and doctors are often overloaded with patients in their daily consultation [12,13].

**Fig 1:**
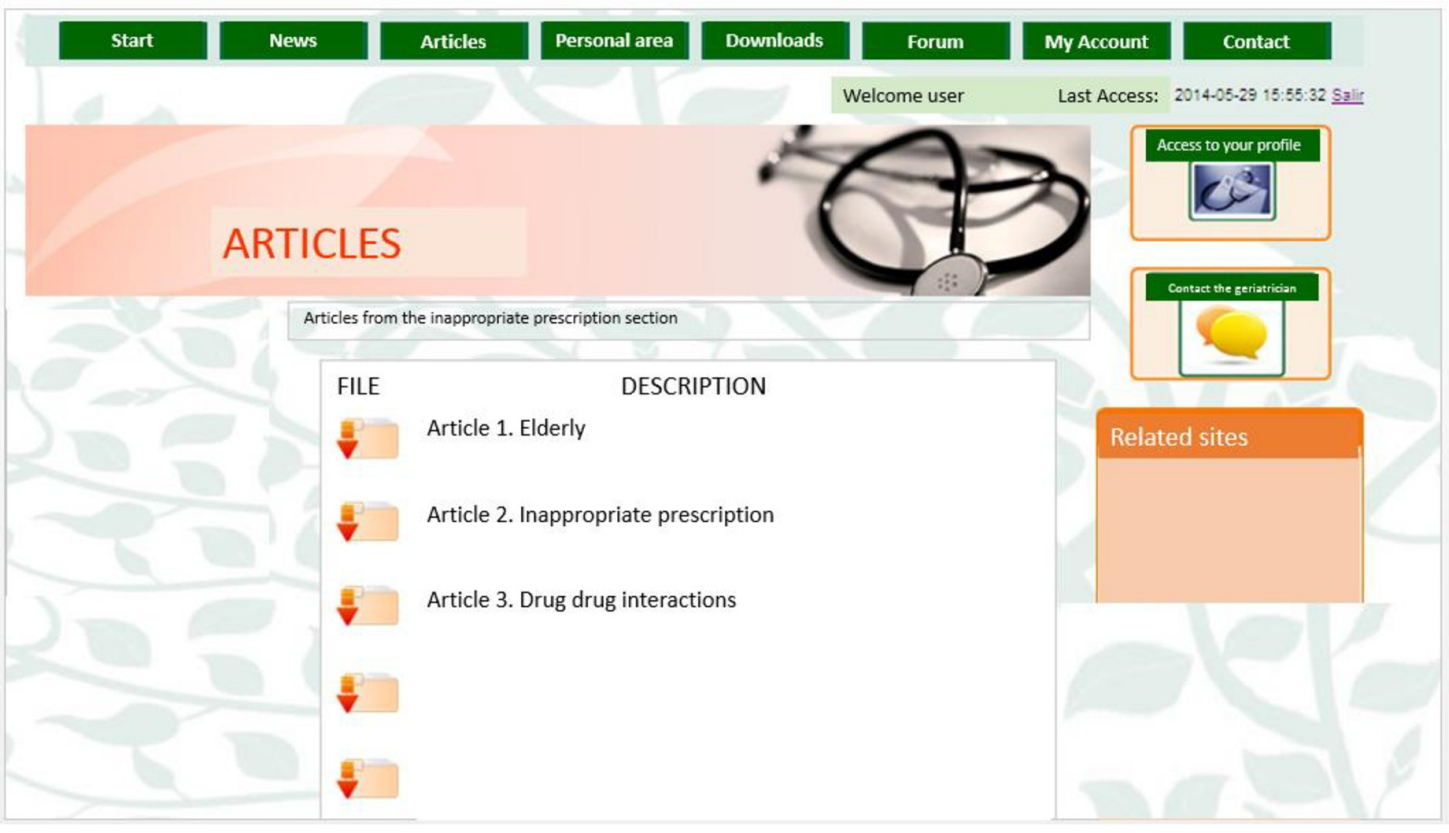
Components of the AGAlink.

**Fig 2:**
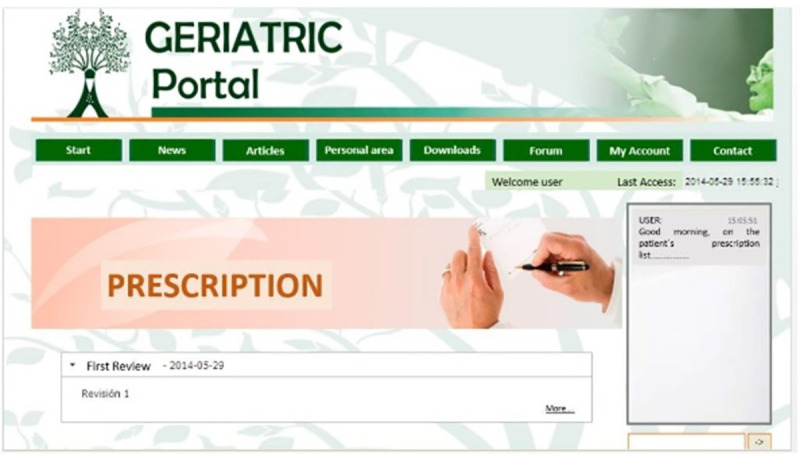
AGAlink screen.

**Fig 3:**
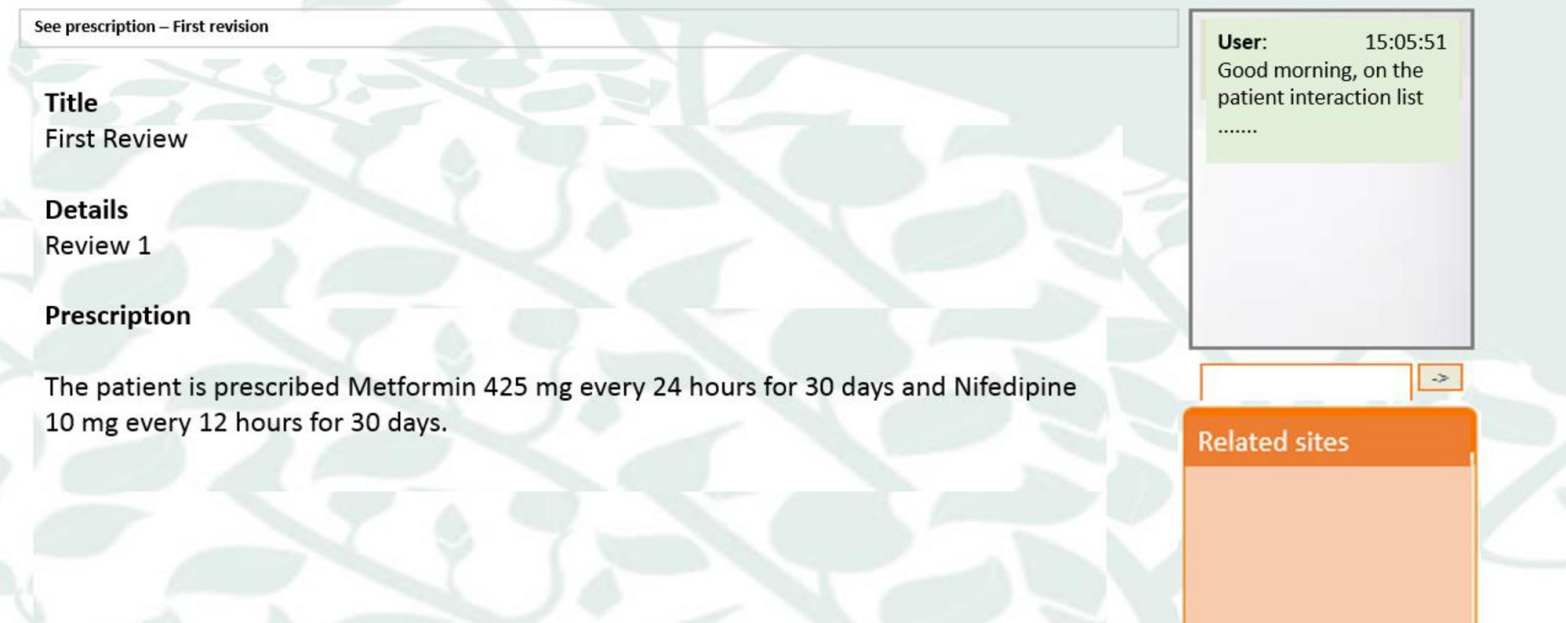
AGAlink example.

For this reason, in this study, an electronic tool called geriatric portal AGAlink was developed to allow primary care physicians to freely decide on the reduction, change or elimination of any medication, for this the portal provided each doctor with the necessary information so they could make the best decision for the benefit of their patients. The components of the AGAlink geriatric portal are shown in Fig 1. Figs 2 and 3 show the screenshot of the geriatric portal, as well as the content that the doctor could see in his personal profile.

The purpose of this study was to carry out a pilot test to know the important points for the acceptance and use of the AGAlink that would allow its improvement and its implementation on daily practice at the first level of care for the prevention of prescription problems among older adults. Identifying how well physicians adopted the recommendations provided by the portal.

## Method

A pilot study was carried out using a semi-structured interview consisted of 16 items to know in depth the preferences, suggestions and comments of family doctors in order to evaluate and optimize the development of a new online asynchronous geriatric program (AGAlink).

### Participants

Two Family Medicine Units (UMF) from the first level of care of the Mexican Social Security Institute (IMSS) were selected, with the same sociodemographic characteristics, all doctors were invited to participate, consent was used to inform the doctor what the research consisted of, subsequently included all adult patients older than 60 years and over, who had a consumption of 4 or more drugs simultaneously by each of the physicians who agreed to participate.

The research protocol was reviewed and approved by the National Commission for Scientific Research, as well as by the IMSS Ethics Commission, with registration number 2011-785-001. To guarantee the anonymity of the participants, the participant’s name was replaced by a pseudonym.

A semi-structured interview was conducted with each of the 31 primary care family physicians who agreed to participate in the study, who had a technological device with internet (telephone, tablet) and who used the internet for at least 10 minutes per day. The semi-structured interview contained open questions that allowed us to explore the use of the online asynchronous geriatric portal called AGAlink and gather information on how this new tool could be implemented in the first level of care for its daily use (Table 1) [14].

**Table 1:** Questions.

• What are the main factors that would motivate you to enter a clinical research study, which consists of an educational intervention using an online geriatric counseling portal? • If your answer is remuneration, which type would you prefer? • Do you consider that the geriatric population is relevant given that in its clinical practice it offers consultation to this population? • Do you work two paid shifts a day? • Are you familiar with using the internet? • Do you have internet service at home? • What time do you go online? • Approximately how long do you connect to the internet? • In general, what day (s) are you most likely to go online? • During your working day, do you have facilities to access the internet? • If you work in another public or private institution, do you have internet access facilities in it? • What are the main limitations to access the internet? • To find relevant information on clinical practice, what sites do you visit? • Given your field of action, which free tool would you like to find on an internet portal? • Reasons why you would not enter an online geriatric counseling portal • Comments and suggestions.

The strategy to carry out the interviews was to start with a series of open questions that helped to address the knowledge about the use of technological tools for therapeutic support, in the search for the identification and reduction of prescription problems within the context of primary or first contact care.

This was with the purpose of identifying the options that the doctor has to access new technologies during their clinical practice and how this facilitates or hinders the selection of treatment, for that we asked the doctor to describe how the best technological tool would be for the patient care, including the information required to be added, their experiences, attitudes and acceptance towards the use of technology as a support tool in their daily practice regarding the treatment of their patients, documenting the resources used during their daily practice and their vision for the future. For each of these elements, we record the frequency of each interviewee.

We also investigated the way in which the doctor got in touch with the asynchronous geriatric portal on the AGAlink line, as well as the barriers and conflicts found and the solutions found in it for their daily actions in front of the treatment for each of their patients. To do this, we start from the assumption that the perspective of others is meaningful and recognizable and can be expressed explicitly [15]. All interviews, which lasted approximately 30 min, were audio-recorded and fully transcribed, in order to have enough information to comply the objectives of the study.

The interviews were conducted by a doctoral student in who was not related to any of the interviewees, and was supervised by a geriatrician specialized in pharmacovigilance, and a computer systems engineer.

It was made clear to each participant that they could freely express their opinion without this being detrimental to them or having any repercussions on their work within IMSS. The interviews were conducted in the office of each of the doctors so that they felt comfortable and with the freedom to talk about their experiences and needs with electronic tools as support for the therapeutic decision of their patients. The established criteria for reporting and publishing qualitative research (COREQ) were followed [16].

#### Analysis

With the information from the interviews, we carry out a thematic analysis of qualitative texts, which is a complementary strategy that focuses on an analysis of the relationships between the different elements of the actors discourses [17]. Where the process of organization and treatment of information was carried out in four large phases.

The first consisted of defining the central issues that were raised with the doctors during the interview. In the second phase, a systematic analysis of the text is carried out, to obtain categories and subcategories [18]. The third phase consists of the formulation of codes and subcodes that allow summarizing the categories and subcategories, and finally the fourth phase consists of the analysis of the information [19], looking for the connections and relationships that allow the interpretation and explanation of the information provided by the interviewed doctors. Any controversy in the analysis was resolved by consensus. The ATLAS program was used to perform the analysis.

## Results

Of the doctors participating in the study, 64% belonged to the female sex, with an average age of 37.8 ± 6.5 years, 93% had a specialty in family medicine. The 31 physicians considered the geriatric population important in their clinical practice.

Acceptance of the intervention AGA link.

In relation to the acceptance of the intervention and its implementation of the use of the AGAlink online asynchronous geriatric portal, as a tool for therapeutic support, 56.7% expressed that they would use the tool and 40% thought that it would allow them to improve their daily practice.

The most important experience was having considered that the use of this tool allowed them to solve their doubts and above all it provided them with new options to solve their prescription problems.

They expressed their satisfaction the use of AGAlink did not consume much time because they did not have to write any information, the person in charge of reviewing the files and completing the information was a pharmacist so that the group of experts could identify the problems of prescription, the doctor was only notified by a message on his cell phone that he had to enter his portal if a problem was identified with his patients to identify which patient had the problem, what type of problem, as well as the options to solve it.

After a while the doctors were already familiar with AGAlink and entered without receiving notification via message, only with the intention of updating their knowledge and consulting their concerns with the group of portal experts through the use of the chat enabled on the portal.

*Being able to enter the portal on any device and at any time encouraged me to use it at first*, *I did not understand its importance, but after the first sessions I discovered its usefulness*, *it allowed me to update myself and consider it a personal learning opportunity*. *(ES 5)*

Some doctors took time to become familiar with the AGAlink tool, so at the beginning the percentage of use was under 20%, but with the passage of time they realized the usefulness and advantages of using the portal.

*At first I did not understand how to use the geriatric portal, nor how it could help me until*, *out of conviction, I started to explore it and discovered that I could update myself*, *learn more about how problems related to the medications that I prescribe to my patients can be avoided… (ES 610)*

In some cases, doctors complained that they did not have access to the internet during their workday, which made it difficult to use AGAlink in their office, so they could only use it at home. They felt that not being able to use it in front of their patient could delay correcting the identified error, making it impractical.

*Waiting for the information of my patients to be reviewed after the therapeutic scheme to follow has been indicated, I consider it will delay the ability to correct the failure*, *I do not feel comfortable, I would prefer that the support was immediate*, *when I am in contact with the patient. (ES 21)*

### Evaluation of the AGAlink

The recommendations provided by the physicians served to update and complement the existing content on the portal. They recognized the importance of updating their knowledge on drug interactions and their adverse effects.

*It is evident that we must use technology to improve clinical practice*. *In our case, treating adults can lead to more prescription problems caused by their own health condition*, *greater number of diseases, etc. (ES 8)*

Acceptance of the AGAlink portal was evidenced, recognizing the usefulness, generating confidence to be used, as well as in the recommendations found in which they were supported to keep in mind what should be taken into account during each prescription.

*Sometimes the workload makes us forget the important points that can avoid complications with the treatment that is prescribed to the patient*, *so it is always useful to have resources such as this portal to remind us and update our knowledge. (ES 6)*

Likewise, they pointed out that sometimes the recommendations or options to modify the treatment were complicated or difficult to carry out, on the other hand, it was suggested that rigorous actions should also be implemented to comply with the observations.

*One of the things that I identified was that some of the recommendations to modify the treatment scheme could not be applied because sometimes the suggested medication was not available*. *(ES 18)*

### Adoption of the recommendations

Most of the doctors made modifications to their therapeutic regimens following the recommendations found in their profile on the portal. They felt that this benefited their patients, and also conditioned them to be more careful when choosing the drug to be prescribed according to the conditions of each patient.

*The fact that the medications were reviewed and the problems identified were pointed out to us was quite good since it made us reflect on the care that should be taken with each patient*, *I followed all the observations, change the medication when indicated*, *modify the dose (I was not careful if my patient had a health problem that altered the metabolism of the drug), (ES 189)*

*I could not make all the changes*, *a patient was upset because I told him that we would change the medications and even reported me to my superior*, *sometimes it was difficult to follow the instructions (ES 13)*

Some physicians did not consider it prudent to change the medication based on their experience and at other times they only temporarily stopped the medication and then prescribed it again.

*I only made some changes, for example they asked me to change a drug for another from the same family*, *or to eliminate a drug that later had to be re-prescribed because it was necessary for the patient. (ES19)*.

In some way, the acceptance of the AGAlink tool was influenced by the knowledge previously acquired by family doctors, as well as by the type of interaction they have with their patients, as a whole they felt that it was useful, that it improved their daily practice and allowed them to expand their knowledge.

### Barriers to adopting recommendations

Barriers and obstacles that physicians faced in order to follow the recommendations were identified through the conversations analyzed (Table 2).

**Table 2:** Barriers to considering the recommendations from the perspective of the family doctor.

Use of the same medicine. Lack of motivation or interest in the recommendations. The basic table of medications as a limitation. Patient attitude. Patient diseases. Accessibility to the AGAlink portal.

In some doctors their customs prevailed before the change since they preferred to keep the medicine because it was the one they always used to treat the condition that their patient presented.

The perception of complexity when modifying the therapeutic scheme was an obstacle for the suggestions proposed to correct the prescription errors identified with AGAlink to be followed.

The lack of motivation and interest to follow the recommendations indicated on the portal were other important factors for making modifications to the treatment scheme.

The main barrier that physicians faced were the drugs included in the institution’s basic drug table, which sometimes did not include the therapeutic option required for change.

The attitude of the patient also represented an important barrier since they refused to change the medication, due to the habit of taking the same drug for a long time.

The patient’s own condition also represented a barrier to the acceptance by the doctor for the change in the therapeutic scheme, his empirical knowledge prevailing, since if he considered that the patient required the drug even knowing that it represented a risk, he would not adopt any modification.

The participation of other specialists in the patient’s therapeutic scheme was a barrier for the doctor to adopt the portal’s recommendations, since he considered it inappropriate to intervene in the specialist’s decision, and on some occasions he had to contact him to inquire about his opinion on the matter.

Using the AGAlink tool outside of office hours represented an important barrier since the doctor sometimes forgot or postponed the suggested indications for the change in the therapeutic scheme.

The use of the online asynchronous geriatric portal gave physicians the possibility of adopting critical thinking, allowing them to make better decisions about their patients’ medication, identifying risks and acquiring skills to reduce them and even know how to manage and avoid them.

In general, the perception of family doctors was that this tool should be incorporated into their daily practice, with the possibility of having the internet to be consulted in their consultation hours in front of patients, which would allow them to optimize and improve the safety of medication for the patient, also considered a good strategy to keep up to date and perceived that it gave them important elements for their personal improvement.

## Discussion

The results show evidence on the importance of incorporating AGAlink into the daily practice of family doctors, since it summarizes important information on the appropriate pharmacological treatment and encourages the doctor to prescribe critically and aware that if the appropriate care can cause a serious problem in their patients.

To reduce the problem of inappropriate prescription, various interventions have been carried out, which have consisted of recommendations from pharmacists, computer alerts, medication review, patient education, avoiding the use of inappropriate medications [20], computerized support systems, services nursing homes, multidisciplinary teams, multifaceted approaches and regulatory policies which have yielded different results [9].

Each of these interventions separately have given different results in relation to the acceptance and modification of the medication by doctors, for example the recommendations made by pharmacists, only had a percentage of 24% of acceptance and modification [20].

Regarding educational interventions, their failure has been evidenced by not generating any change in the actions of the doctor, persisting inappropriate prescription among their patients [9,21].

In primary care centers where the computerized alert system is used, 91.2% of physicians ignore them, but 8.8% result in a change in drug prescription [22]. Another study on electronic medical alerts indicates that for these to be convenient, there must be an approval by doctors for their use, in addition, doctors must have access to the appropriate information to make a decision [23].

However, none of these studies have evidenced the beliefs, experiences, attitudes and barriers expressed by physicians regarding this type of intervention.

In this study, it was possible to get family doctors to make changes in medications, dose or frequency of doses. Following the recommendations set out in the AGAlink portal, surpassing the results observed in other studies [12,24].

The use of technology in medicine is being increasingly used [25]. The portal content was ordered, helping physicians to locate what they need to update their knowledge of prescription problems without leaving it. Among the advantages that the doctors pointed out to accept the use of the AGAlink was that they could access it as long as they had an internet connection, the information was available immediately, it was easy to access, the information was presented in a simple way that made it easier for them to make a decision, as well as that they could exchange information [26].

### Future implications for the use of AGAlink

For the implementation of the tools for therapeutic support such as AGAlink, more than one element must be incorporated to be successful in this case, the geriatric portal was not only based on the use of an electronic tool but also involved the participation of the pharmacist, the geriatrician, as well as other educational tools to show doctors how the use of this strategy benefits and allows them to reduce the workload and improve their daily practice.

At the first level of care, it is necessary to implement strategies that allow the identification and reduction of prescription problems among the adult population in order to reduce the complications and expenses that are generated as a result of the administration of a wrong therapeutic scheme. However, acceptance by medical personnel will not be easy, given their custom, beliefs and preferences. On the other hand, the electronic medical record must be improved to guarantee that the lack of data does not indicate the possibility of making mistakes in the identification of prescription problems. This could compromise the reliability of the portal. Finally, the AGAlink can only be used in real time when health systems are connected to the internet.

### Strengths and weaknesses of the study

Family doctors who agreed to participate were interviewed, which could condition the results since they could be more interested in using the AGAlink as a tool in their daily practice. However, doctors of different ages, sex, and seniority were interviewed, which allowed for a wide diversity of points of view and experiences. The interviewed doctors had internet and an electronic device with their own internet access, so the results could be generalized to other similar settings.

The experience with the device was one year, so we could not assess the long-term results. To eliminate possible biases in the results, the interviewers and the information captor were not directly involved in the project.

### Conclusion

The experiences and attitudes of the doctors conditioned the acceptance of the AGAlink tool, in daily practice, there was a favorable response to the content of the geriatric portal on prescription problems and their prevention, encouraging the family doctor to self-criticize and reflect on this problem of health.

However, it was also the experience, beliefs and attitudes of the doctors that conditioned the barriers to modify the therapeutic scheme even after the prescription problem had been notified, the doctor considered that the medicine was necessary, refusing to change it, that the option was not available and its change would become complex, or left the change of the medication or dose in the hands of another specialist.

Given the positive attitude towards the implementation and use of AGAlikn in daily practice among family doctors, it would be worth knowing what the cost effectiveness of its implementation in the first level of care would be.
